# Concentrated Meat Foods

**Published:** 1911-09-09

**Authors:** 


### DIETETICS.

### CONCENTRATED MEAT FOODS.

"Vegetarian" enthusiasts are usually only too eager to point out that "a pound of lentils contains far more nourishment than a pound of meat"—a statement which is very far indeed from being fact. Unfortunately, however, the public, unacquainted with the essentials of dietetics, is not in a position to detect the fallacy in the assertion, and the vegetarian himself is often quite unaware of it. When we have reduced the argument to a logical basis we find that the vegetarian states it thus: "A pound of lentils contains a larger quantity of proteid than a pound of steak; since it contains more proteid it has a relatively higher caloric value, and is therefore a more nourishing food." The fallacies here are two. The premiss presupposes the similarity of lentils and meat—the former a dried and concentrated product, the latter containing three parts water—a presupposition which is inadmissible. The conclusion takes it for granted that the only test of the value of an article of diet as a food is its proteid content and its caloric value—a conclusion which no sane dietician can subscribe to. It is now well known that there are important differences between animal and vegetable proteids, and that the assimilability and digestibility of meat albumin are greater than those of vegetable albumins. Taking the vegetarian's own comparison—admittedly not a reasonable one, since the vegetable food is concentrated—the nutritive value of a pound of lentils lies not in the theoretical caloric of the total proteid content (nearly 1,700 C.), but only in the real caloric of the total assimilable proteid—a far different thing in dietetic practice.

We instance this comparison, erroneous as it is, to show the fallacy of arguing from wrong premisses, and at the same time to point out the dietetic value of concentrated meat foods. By desiccation and appropriate preparation, the water and fat may be removed from meat, and the proteid content concentrated down to almost the historic thimbleful which was all that remained of the Marquis de Cussy's hundred hams. Such concentrated meat preparations are too little known to the public, which has not yet learned that raw meat has its uses on occasions. We consume large quantities of beef teas and meat essences, the food value of which is comparatively low, but we do little to test the value of the various forms of concentrated meat now on the market.

There are many varieties, and the dietician who wishes to experiment with this form of food has a fairly large choice. The solid forms are to be found in the Russian and Finland hams and tongues, thoroughly dried, and more or less deprived of fat; in the South African "biltong," and in the Oriental dried spiced lamb and goats' flesh. All these forms have a high nutritive value, since not only is their percentage of proteid very large, but nearly the whole (99 per cent.) of this proteid is digested and absorbed. Finely scraped, or thinly cut and partaken of in moderation, such dried meat is nourishing, palatable, and very easily assimilated. Unfortunately it is an uneconomical food which is comparatively difficult to obtain, and we have to make a selection from the powdered preparations.

Of these we know none that can surpass the Pemmican and Emergency Ration manufactured by the Bovril Company. Pemmican itself is the dried and pounded flesh of the American bison. Bovril is the powdered flesh of well-fed and carefully-killed cattle, dried and prepared by a special process. It is a brown-grey powder, possessing a pleasant meaty smell, and consists roughly of 88 parts proteid, 6 parts fat, and 6 parts moisture and salts. The Emergency Ration is practically Pemmican which has been enriched by the addition of fat and carbohydrates; but although its caloric value (owing to the greater percentage of fat) is higher, it is not such a generally suitable concentrated food, since the proteid that it contains appears to be less easily digested than that of Pemmican. Eaten as an addition to a bread-and-butter sandwich, as a thickening to soups and gravies, an ingredient in vegetable stews and in bread gruels, such a concentration as Pemmican should prove a very valuable addition to the dietary, and it is particularly suitable for the invalid and convalescent, owing to the ease with which the proteid is digested. Few things can surpass as an emergency luncheon a Pemmican sandwich and a glass of stout—such a combination is easily digested, in the majority of cases easily assimilated, and palatable in the extreme. Comparisons are odious, but we may mention that in the course of a series of dietetic experiments we tested the above combination against the ordinary quick lunch consisting of a hard-boiled egg, a glass of milk, and a roll and butter. The Pemmican lunch had left the stomach within 3½ hours; four hours after the egg and milk lunch examination of the stomach contents showed that gastric digestion was not yet complete. The total food value of the two lunches was almost identical. The use of concentrated meat foods may be objected to as likely to give rise to constipation and to throw a strain on the eliminating organs. That is an objection which the practitioner must bear in mind when suggesting Pemmican or any form of dried meat; it may be overcome by suitably combining the meat powder with food sparers or with foods of a low proteid content. This is best done in the form of vegetable stews—a method which deserves wider recognition than it has yet obtained. The addition of Pemmican to a vegetable broth makes a very nourishing and palatable beverage, far superior to the ordinary broths so often served up, since the actual proteid of the meat is present in the soup. The only difficulty is that at present the Bovril preparations of dried meat, as distinct from the meat extracts, are comparatively little known, and that they are somewhat highly priced. In view of the fact that they are also very highly concentrated, these preparations may be regarded as economical foods which are well worth the practitioner's attention.

